# Chlorophyll Composition, Chlorophyll Fluorescence, and Grain Yield Change in *esl* Mutant Rice

**DOI:** 10.3390/ijms19102945

**Published:** 2018-09-27

**Authors:** Weiwei Lin, Xiaodong Guo, Xinfeng Pan, Zhaowei Li

**Affiliations:** 1 College of Life Sciences, Fujian Agriculture and Forestry University, Fuzhou 350002, China; weiweilin@fafu.edu.cn; 2 College of Crop Science, Fujian Agriculture and Forestry University, Fuzhou 350002, China; 3157614046@m.fafu.edu.cn (X.G.); 3165006035@m.fafu.edu.cn (X.P.); 3 Institute of Crop Science, College of Agriculture and Biotechnology, Zhejiang University, Hangzhou 310058, China

**Keywords:** leaf senescence, Chl fluorescence, photosynthesis, rice (*Oryza sativa* L.)

## Abstract

To evaluate the effect of changes in chlorophyll (Chl) composition and fluorescence on final yield formation, early senescence leaf (*esl*) mutant rice and its wild-type cultivar were employed to investigate the genotype-dependent differences in Chl composition, Chl fluorescence, and yield characteristics during the grain-filling stage. However, the temporal expression patterns of key genes involved in the photosystem II (PSII) reaction center in the leaves of two rice genotypes were analyzed by quantitative real-time polymerase chain reaction (qRT-PCR). Results showed that the seed-setting rate, 1000-grain weight, and yield per plant remarkably decreased, and the increase in the 1000-grain weight during the grain-filling stage was retarded in *esl* mutant rice. Chl composition, maximal fluorescence yield (Fm), variable fluorescence (Fv), a maximal quantum yield of PSII photochemistry (Fv/Fm), and net photosynthetic rate (*Pn*) in *esl* mutant rice considerably decreased, thereby indicating the weakened abilities of light energy harvesting and transferring in senescent leaves. The *esl* mutant rice showed an increase in the minimal fluorescence yield (F_0_) and 1 − Fv/Fm and decreases in the expression levels of light-harvesting Chl *a*/*b* binding protein (*Cab*) and photosystem II binding protein A (*PsbA)*, *PsbB*, *PsbC*, and *PsbD* encoding for the reaction center of the PSII complex during the grain-filling stage. These results indicated the PSII reaction centers were severely damaged in the mesophyll cells of senescent leaves, which resulted in the weakened harvesting quantum photon and transferring light energy to PSI and PSII for carbon dioxide assimilation, leading to enhanced heat dissipation of light energy and a decrease in *Pn*.

## 1. Introduction

As a staple food, rice (*Oryza sativa* L.) is one of the most important cereal crops in the world because it feeds nearly a half of the world’s population, especially in Asia [1]. Rice grain yield is highly dependent on the photosynthetic assimilation of leaves during the grain-filling stage. Previous studies have shown that 60% to 80% of nutrients required for rice grain filling are contributed by the photosynthesis of source leaves during the grain-filling period [2,3]. The rest of the nutrients are made up of remobilized carbohydrates that are temporarily stored in culms and leaf sheaths before the heading stage [4]. The photosynthetic intensity and durability of the source leaves are essential for grain filling in the rice plant. Maintaining the vitality of functional rice leaves during the grain-filling stage guarantees carbohydrate synthesis and increased yield [5]. The final grain yield can increase by 1% if the leaf lifespan can be prolonged for 1 day during the grain-filling stage for the rice plant [6]. However, leaf senescence is one of the main factors that restrict the yield and quality of grain in hybrid rice varieties. Leaf senescence often occurs prematurely under severe environmental stresses during the grain-filling stage. Premature leaf senescence shortens the photosynthetic duration and reduces the amount of assimilation substance that originates from photosynthesis, thereby retarding nutrient translocation from source leaves to developing grains during the grain-filling stage, resulting in incomplete grain setting, and reducing the final grain yield [7,8].

Leaf senescence is the final phase of development that culminates in the death of the cell, tissue, and organ. A positive purpose of leaf senescence is to remobilize valuable nutrients from older leaves for recycling in grain filling during the grain-filling stage [9]. However, a consequence of leaf senescence is that photosynthesis declines with the degradation of chlorophylls (Chl) and proteins involved in photosynthetic reactions, indicating a problem in the efficient operation of photosynthesis [10]. The decrease in photosynthesis is associated with the structural changes in thylakoid membranes, which are related to the disintegration of the grana and damage of the stromal thylakoids [11]. In the membranes of the thylakoids, the light-harvesting complexes consist of CP43, CP47, D1, D2 proteins, and Chl; they are mainly involved in the harvesting and transportation of light energy and play a primary role in the photosynthetic reaction [12]. During leaf senescence, the reduction of photosynthesis is associated with the breakdown of protein components and Chl content [13]. In addition, Chl fluorescence is often used in biological research as an indicator of photosynthetic regulation and plant responses to various stresses [14,15]. The fluorescence parameters are closely correlated with the function of photosystem II (PSII), and the photoinhibition of PSII is easily sensed by a reduction in the dark-adapted ratio of variable to maximum Chl fluorescence [16]. Analysis of the Chl fluorescence parameters can provide valuable information on the photosynthetic apparatus [17]. Stefanov et al. [18] found that high light stress reduces the content of pigments and efficiency of photochemical energy conversion and inhibits the maximum and effective quantum yields of PSII photochemistry, resulting in a decrease in the photochemical quenching and photosynthetic rates in two hybrid lines. During leaf senescence in wheat, the apparent quantum yield of net photosynthesis decreased; the maximal efficiency of PSII photochemistry slightly decreased and led to the down-regulation of the biological function of the PSII apparatus, followed by reduced net photosynthesis in senescent leaves [19]. Wingler et al. [20] investigated the leaf senescence of *Arabidopsis* and revealed that nonphotochemical quenching and minimum fluorescence (F_0_) increase in the tips of inner rosette leaves during the early stage of leaf senescence. However, little is known regarding the involvement of pigment composition and Chl fluorescence parameters in the leaf senescence of rice plants during the grain-filling stage. In addition, the changing mechanism of the core components of the PSII reaction center has yet to be elucidated during leaf senescence.

Therefore, the current study aimed to evaluate the changes in Chl composition and fluorescence and compare the genotype-dependent differences in the efficiency of the photosynthetic apparatus in the leaves of early senescence leaf (*esl*) mutant and its corresponding wild-type cultivar during the grain-filling stage. The genotype-dependent differences in the temporal expression patterns of key genes involved in the PSII core reaction center in the leaves were analyzed by quantitative real-time polymerase chain reaction (qRT–PCR).

## 2. Results

### 2.1. Genotype-Dependent Differences in the Yield Properties and Grain Filling

**Figure 1:**
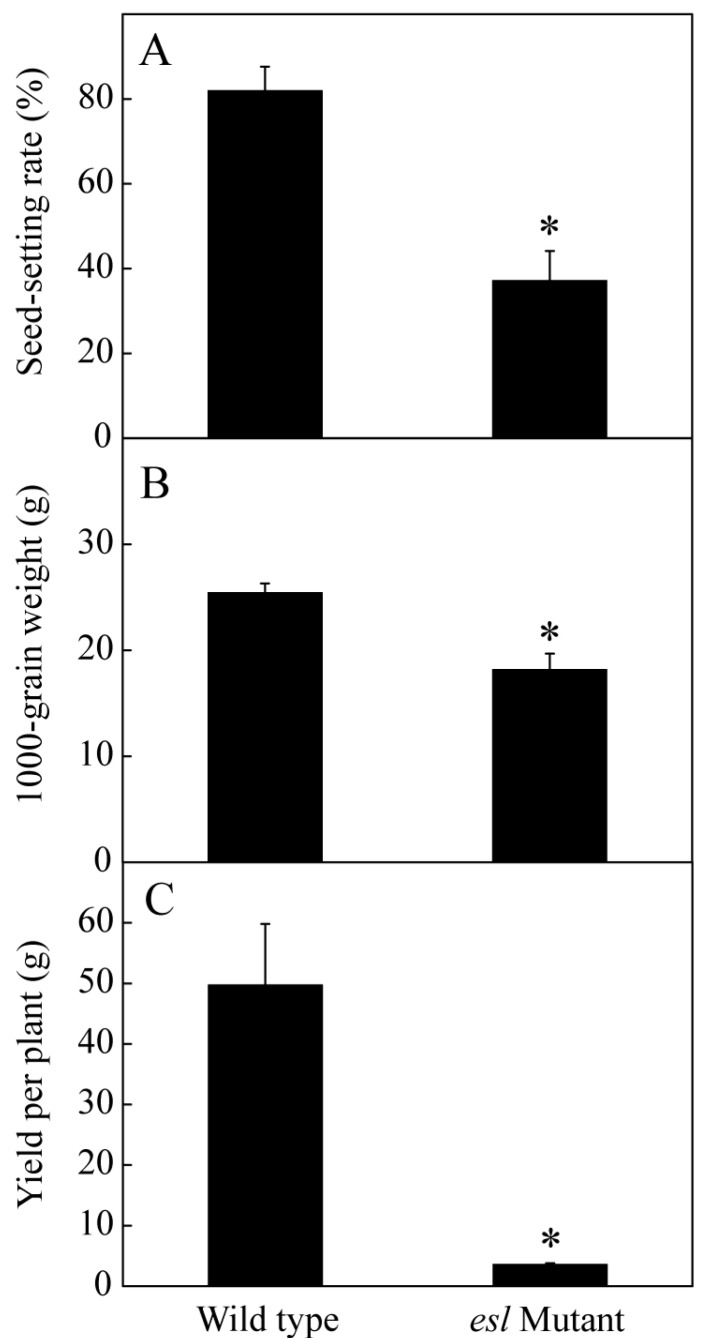
Genotype-dependent differences in the (**A**) seed-setting rate, (**B**) 1000-grain weight, and (**C**) yield per plant for the two rice genotypes. Vertical bars represent standard errors (*n* = 3). The asterisks represent significant differences between the early senescence leaf (*esl*) mutant and its wild-type cultivar (* *p* < 0.05).

**Figure 2:**
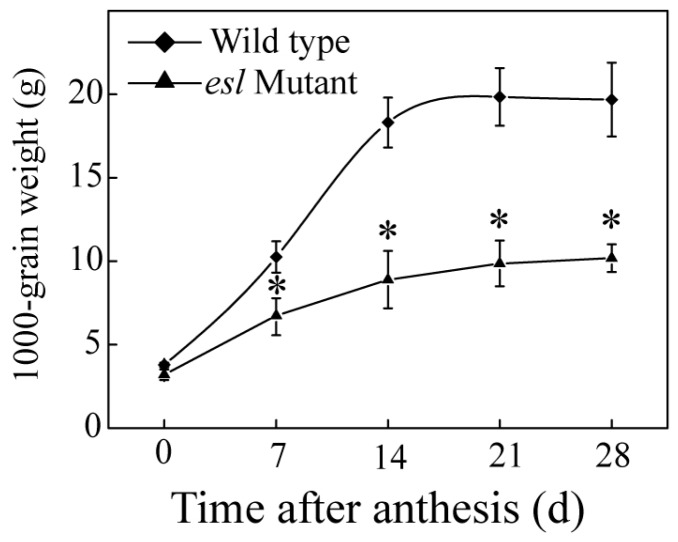
Genotype-dependent differences in the temporal increases of 1000-grain weight for the two rice genotypes during the grain-filling stage. Vertical bars represent standard errors (*n* = 3). The asterisks represent significant differences between the *esl* mutant and its wild-type cultivar (* *p* < 0.05).

To compare the genotype-dependent differences in the yield properties, the seed-setting rate, 1000-grain weight, and grain yield per plant were investigated for the *esl* mutant rice and its wild-type after harvesting. Figure 1 shows a significant difference between the two rice genotypes for the three properties. The seed-setting rate of the *esl* mutant rice was only 37%, which was significantly lower than that of the wild-type (82%; Figure 1A). The 1000-grain weight of the *esl* mutant rice was 17 g, which was also lower than that of the wild-type (nearly 25 g; Figure 1B). In addition, the *esl* mutant rice achieved 3.6 g of grain yield for each plant and showed a considerably reduced yield compared with the wild-type (49.7 g; Figure 1C). These results suggested that the yield properties of the *esl* mutant rice deteriorated. The dynamic changes in the 1000-grain weight during the entire grain-filling stage showed that the 1000-grain weight increased slowly for the *esl* mutant rice but increased rapidly for the wild-type. Moreover, the 1000-grain weight of the *esl* mutant rice was significantly lower than that of the wild-type after 7 days of grain filling (Figure 2). This result suggested the decelerating grain-fitting rate and weakening seed-setting rate in the *esl* mutant rice.

### 2.2. Genotype-Dependent Differences in Leaf Characteristics and Photosynthetic Performance during the Grain-Filling Stage

**Figure 3:**
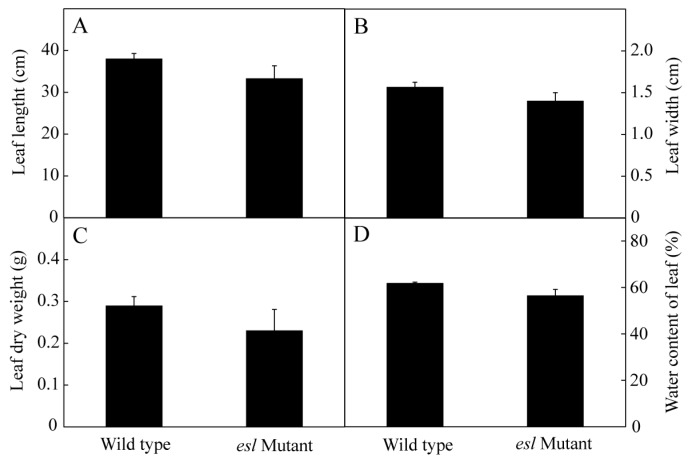
Comparisons of the (**A**) leaf length, (**B**) leaf width, (**C**) leaf dry weight, and (**D**) water content of leaves between the *esl* mutant rice and its wild-type cultivar. Vertical bars represent standard errors (*n* = 3).

**Figure 4:**
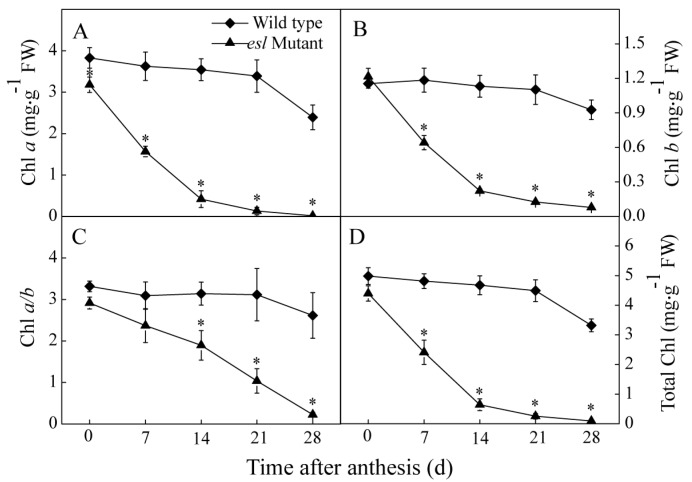
Genotype-dependent differences in (**A**) chlorophyll (Chl) *a*, (**B**) Chl *b*, (**C**) Chl *a*/*b*, and (**D**) total Chl for the flag leaves of the two rice genotypes during the grain-filling stage. Vertical bars represent standard errors (*n* = 3). The asterisks represent significant differences between the *esl* mutant and its wild-type cultivar (* *p* < 0.05).

As the most important source organ, the flag leaf plays an essential role in grain filling during the grain-filling stage. The genotype-dependent differences in the flag leaf length, width, dry weight, and relative water content were investigated at the heading stage. Figure 3 shows no significant differences in the traits between the two rice genotypes. However, significant differences in Chl parameters were detected between the two rice genotypes (Figure 4). The contents of Chl *a* and *b* and the total Chl content in the *esl* mutant rice leaves rapidly decreased and were significantly lower than those of the wild-type rice leaves during the grain-filling stage (Figure 4A,B,D). The Chl *a*/*b* value in the *esl* mutant rice leaves showed a linear decline and was significantly lower than that of the wild-type rice leaves (Figure 4C), thereby suggesting that degradation of Chl *a* was greater than that of Chl *b* in the *esl* mutant rice leaves.

**Figure 5:**
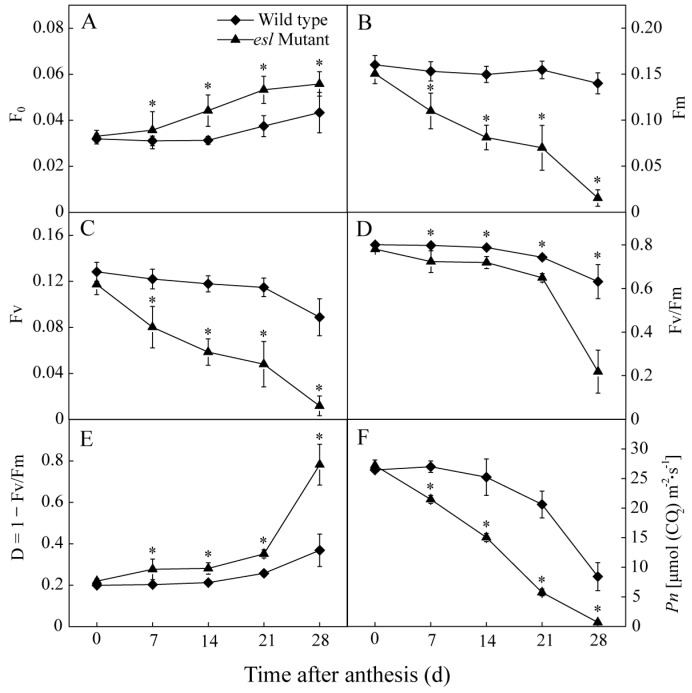
Genotype-dependent differences in the temporal changes in (**A**) minimal fluorescence yield (F_0_), (**B**) maximal fluorescence yield (Fm), (**C**) variable fluorescence (Fv), (**D**) Fv/Fm, (**E**) 1 − Fv/Fm, and (**F**) net photosynthetic rate (*Pn*) for the flag leaves of the two rice genotypes during the grain-filling stage. Vertical bars represent standard errors (*n* = 3). The asterisks represent significant differences between the *esl* mutant and its wild-type cultivar (* *p* < 0.05).

In addition, the photosynthetic performance was assessed by determining the Chl fluorescence parameters and net photosynthesis rate. Figure 5 shows that F_0_ in the *esl* mutant rice leaves increased gradually and became higher than that of the wild-type leaves after 7 days in the grain-filling stage (Figure 5A). By contrast, maximal fluorescence yield (Fm) in the *esl* mutant rice leaves decreased gradually and was significantly lower than that of the wild-type leaves during the same period (Figure 5B). In comparison with the wild-type rice, the *esl* mutant rice leaves showed a significant decrease in the variable fluorescence (Fv) and Fv/Fm values and a significant increase in 1 − Fv/Fm during the grain-filling stage (Figure 5C–E), thereby suggesting that the reducing primary quinone acceptor (Q_A_) reduction and quantum yields of PSII photochemistry and increasing heat dissipation of light energy occurred during the photosynthesis of the *esl* mutant rice leaves. Moreover, the *esl* mutant rice leaves exhibited a linear decrease in net photosynthetic rate (*Pn*), which was significantly lower than the wild-type rice leaves after 7 days in the grain-filling stage (Figure 5F).

### 2.3. Genotype-Dependent Differences in the Expression of Cab and Key Genes Encoding for the Reaction Center of the PSII Complex

**Figure 6:**
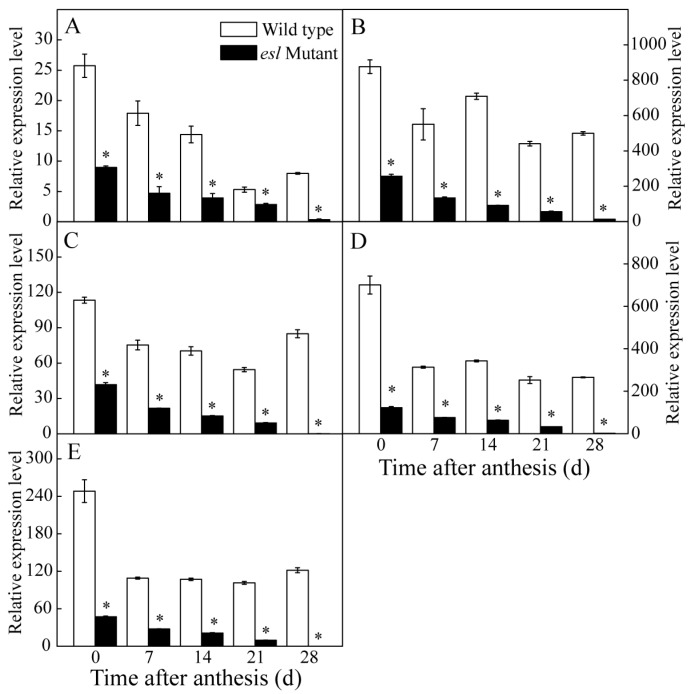
Comparison of the temporal expression patterns of (**A**) light-harvesting Chl *a*/*b* binding protein (*Cab)*, (**B**) photosystem II binding protein A *(PsbA*), (**C**) *PsbB*, (**D**) *PsbC*, and (**E**) *PsbD* in the flag leaves between the two rice genotypes during the grain-filling stage. Vertical bars represent standard errors (*n* = 3). The asterisks represent significant differences between the *esl* mutant and its wild-type cultivar (* *p* < 0.05).

In photosynthesis, the light-harvesting Chl (LHC) *a*/*b* binding protein (Cab) is involved in harvesting quantum photon and transferring light energy to the PSI and PSII for carbon dioxide assimilation. A genotype-dependent difference in the expression of *Cab* was determined during the grain-filling stage. Figure 6A shows that the expression levels of *Cab* in the leaves of the two rice genotypes decreased gradually during the entire grain-filling stage. Compared with the wild-type rice, the *esl* mutant rice showed a significantly lower expression pattern of *Cab* in the leaves during the grain-filling stage. In addition, four key genes encoding for the reaction center of the PSII complex were detected for the two rice genotypes. As shown in Figure 6B–E, the expression levels of photosystem II binding protein A (*PsbA*), *PsbB*, *PsbC*, and *PsbD* in the *esl* mutant rice leaves decreased gradually after anthesis and were significantly lower than those of the wild-type rice leaves during the entire grain-filling stage. These results indicated the weakened ability of light harvesting and energy transfer and reduced electron transport through the reaction center of PSII in the *esl* mutant rice leaves during the entire grain-filling stage.

## 3. Discussion

Leaf senescence is an important trait that influences crop yield and quality. In cereal crops, such as barley, wheat, and rice, leaves are the major source of nutrients for the developing grains during the grain-filling stage. Delaying leaf senescence, particularly of the flag leaf, will result in a significant increase in crop yields [21]. In recent years, the stay-green traits have been considered in breeding practice to enhance stress resistance and increase grain yield [22]. By contrast, early leaf senescence often leads to a severe decrease in crop productivity during the grain-filling stage [23,24]. In this study, early senescence in the *esl* mutant rice showed significant decreases in the seed-setting rate, 1000-grain weight, and yield per plant relative to the wild-type rice (Figure 1). The temporal accumulation of the 1000-grain weight in the *esl* mutant rice was significantly lower than that of the wild-type rice during the grain-filling stage (Figure 2), suggesting that the amounts of nutrients transported in the developing grains were reduced for the *esl* mutant rice during the whole grain-filling stage. Therefore, the dynamic filling of the developing grains during the grain-filling stage was apparently weakened in the *esl* mutant rice in terms of early leaf senescence, which finally contributed to the reduced seed-setting rate, 1000-grain weight, and yield per plant in the *esl* mutant rice when harvested. The present demonstration also agreed with a previous conclusion that the reduced nutrient translocation and grain-filling rates due to early source leaf senescence are one of the major reasons that result in insufficient grain filling and severe loss of grain yield in cereal crops [25,26].

Plant leaf is an important organ of photosynthesis. In particular, the uppermost flag leaf has a crucial role in supplying photosynthates to the developing grains [27,28]. Previous studies have demonstrated that grain yield characteristics are positively correlated with flag leaf traits [29]. Faisal [30] investigated the relationship between flag leaf characteristics and grain yield in three cereal crops (oat, wheat, and rice) and discovered that grain yield is significantly and positively correlated with leaf area and fresh weight of flag leaf. In the present study, no significant differences were observed for leaf length, leaf width, leaf dry weight, and water content in the flag leaves between the *esl* mutant and its corresponding wild-type rice genotypes (Figure 3). The morphological characteristics of the flag leaf were not the principal reason for the loss of yield in the *esl* mutant rice.

On the other hand, the yield formation of cereal crops is also closely related to the Chl content of the flag leaf during the grain-filling stage, and abundant Chl in the source leaf is required for the biosynthesis of photosynthates [31]. The most salient feature in the early senescent leaf is the yellowish phenotype due to Chl degradation during chloroplast decomposition, which results in reduced photosynthetic activity [32]. In the present study, the flag leaf of the *esl* mutant rice displayed significant decreases in Chl *a*, Chl *b*, Chl *a*/*b*, and total Chl compared with the wild-type rice during the grain-filling stage (Figure 4). The decrease in the Chl content due to early leaf senescence was likely the principal reason that resulted in the loss of final yield. The significant decrease in Chl *a*/*b* for the early senescent leaf suggested that the degradation rate of Chl *a* was faster than that of Chl *b* during leaf senescence. Simeonova et al. [33] reported similar results and observed a more rapid decrease in the Chl *a* content than in the Chl *b* content during leaf senescence for rice and wheat. Chl *a* was possibly highly sensitive to reactive oxygen species that accumulated in mesophyll cells during leaf senescence [34]. Another possible reason is that the degradation of Chl *b* was more complicated than that of Chl *a* because the degradation of Chl *b* must first be converted to Chl *a* via 7-hydroxymethyl Chl *a* [35]. In addition, the *esl* mutant rice leaves showed an increase in F_0_ (Figure 5A) and decrease in Fm (Figure 5B) and Fv (Figure 5C) during the entire grain-filling stage, indicating the weakened abilities of light energy harvesting and transfer in mesophyll cells of senescent leaves. These effects were due to the reduced Chl *a* and Chl *b* levels, because Chl *a* is responsible for energy excitation and transfer in photosynthesis, and Chl *b* is considered an antenna Chl and adopts an essential role in light energy capture and energy aggregation [36].

Increasing evidence has demonstrated that Chl takes a central role in photosynthesis by forming complexes with thylakoid-membrane proteins such as PSI, PSII, and the cytochrome b6f complex [32,37]. In this study, the *esl* mutant rice showed a significant decrease in Fv/Fm value during the grain-filling stage (Figure 5D). The expression levels of *PsbA*, *PsbB*, *PsbC*, and *PsbD* encoding for the core reaction center of PSII complex were significantly lower for the *esl* mutant rice than those for the wild-type rice during the entire grain-filling stage (Figure 6B–E). Thus, the structure of the PSII complexes inlaid in the thylakoid membrane was severely damaged, and the PSII apparatus lost some biological function in senescent leaves. The destroyed thylakoid structure was also observed in senescent rice leaves by electronic super-microscopy [38]. The severely damaged PSII reaction centers resulted in the reduced Q_A_ reduction and quantum yields of the PSII photochemistry. However, the significantly decreased expression of *Cab* in senescent leaves (Figure 6A) suggested the weakened harvesting of quantum photon and transfer of light energy to the PSI and PSII for carbon dioxide assimilation. Subsequently, a large amount of light energy dissipated in the form of heat energy in mesophyll cells of senescing leaves. This phenomenon was supported by the result of a significant increase in 1 − Fv/Fm in the *esl* mutant rice leaves (Figure 5E), demonstrating the increased heat dissipation of light energy in mesophyll cells of senescent leaves. Therefore, a significant decrease in *Pn* for the *esl* mutant rice (Figure 5F) was a necessary result of the decreased harvesting and utilization efficiency of light energy in senescent leaves.

## 4. Materials and Methods

### 4.1. Plant Material

**Figure 7:**
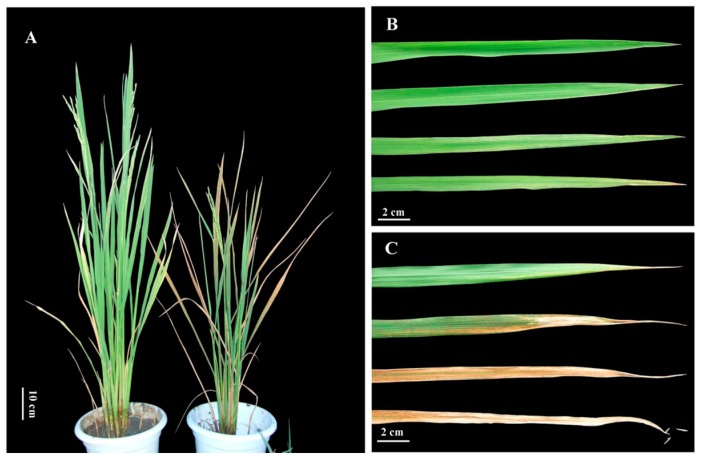
Comparisons of plant morphological phenotypes (**A**) between *esl* mutant (**right**) and its corresponding wild-type cultivar (**left**) at the heading stage. The flag leaves of *esl* mutant (**B**) and wild-type cultivar (**C**) sampled over 8-day intervals from anthesis to 24 days after anthesis.

Samples of *esl* mutant rice with the early leaf senescence phenotype were obtained from the mature seeds of gamma-irradiated cultivated rice “Fu142” (*Oryza sativa* L. *indica*), which was acquired from Institute of Crop and Nuclear Technology Utilization, Zhejiang Academy of Agricultural Sciences in Hangzhou, Zhejiang Province, China. Stable phenotype selection was performed from the mutation (M) 2 to 8 generations through successive self-pollination. The *esl* mutant rice plant did not show obvious phenotypic abnormalities between the seedling and tillering stages. During the late tillering stage, lesions began to appear on the tips of the lower leaves of the *esl* mutant rice, and brown lesions gradually became exacerbated and expanded to cover the whole leaf blade. However, the topmost two fully expanded leaves and central leaf of plant retained their normal green appearance (Figure 7A). After the flowering stage, the flag leaf of the *esl* mutant rice plant began to display senescence symptoms, and the brown lesions gradually spread from the tip down to the whole leaf blade during the grain-filling stage until the leaf completely became withered at nearly 25 days after anthesis (Figure 7B). By contrast, the wild-type rice plant retained its normal green appearance during the same period (Figure 7C).

### 4.2. Field Study

The experiment was conducted in 2017 at the experimental field of the Fujian Agriculture and Forestry University in Fuzhou, Fujian Province, China. The soil type at the field was periodically waterlogged paddy soil with 1.69 g/kg total N, 24.5 mg/kg available P, and 103.7 mg/kg exchangeable K. The completely randomized field plots were arranged with three replications for each genotype. Rice seeds were presoaked in water for 24 h and then germinated at 37 °C for 24 h in the dark. The germinated seeds were sown in a seedling nursery on April 15 and transplanted on May 10. Each field plot was set in 10 × 12 rows and spaced at 18 cm × 18 cm with one rice seedling for each hill. Field plot management was implemented based on the local cultivation mode. At the full heading stage, 100–120 plants with the same flowering day were randomly selected and tagged for each rice genotype. From the beginning of the grain-filling stage, Chl fluorescence parameters and photosynthetic rate of the flag leaves of the tagged rice plants were measured over 7-day intervals at 11:00 a.m. The flag leaves were also sampled in the same period with three independent biological replications. Some fresh leaf samples were used for the determination of photosynthetic pigments, and other fresh samples were immediately frozen in liquid nitrogen and kept at −80 °C for gene expression analysis. Fifteen panicles were harvested over 7-day intervals, and the grains were threshed by hand and dried in an oven. The 1000-grain weight was weighted over 7-day intervals during the grain-filling stage. At the maturity stage, the seed-setting rate, 1000-grain weight, and yield per plant were investigated for each rice genotype.

### 4.3. Determination of Photosynthetic Pigments

Photosynthetic pigment contents were measured as described by Lichtenthaler [39]. A leaf sample (0.2 g) was ground and soaked in 95% (*v*/*v*) alcohol. The sample was stored in a dark place for approximately 24 h until the color of the sample changed to white. After centrifugation, the absorbance of the supernatant was measured at 470, 649, and 665 nm by a spectrophotometer (UV-2450, Shimadzu, Kyoto, Japan).

### 4.4. Measurement of Chl Fluorescence Parameters and Net Photosynthetic Rate (Pn)

Chl fluorescence parameters were measured using a steady-state gas-exchange system with an integrated fluorescence chamber head (LI-6400-40, LI-COR, Lincoln, NE, USA). Six plants for each rice genotype were adapted in the dark for 20 min and then irradiated with weak light (0.1 μmol [photon] m^−2^·s^−1^). The minimal fluorescence yield of the dark-adapted state (F_0_) was measured, and the maximal fluorescence yield of the dark-adapted state (Fm) was measured by applying a saturating pulse light (3000 μmol [photon] m^−2^·s^−1^). Variable fluorescence (Fv) was calculated as Fv = Fm − F_0_, and the maximal quantum yield of PSII was calculated as Fv/Fm = (Fm − F_0_)/Fm, which was used to assess the photochemical efficiency of PSII. The heat dissipation of light energy was calculated as D = 1 − Fv/Fm. *Pn* in flag leaf was measured from 11:00 to 12:00 a.m. using an LI-6400 portable photosynthesis system (LI-COR, Lincoln, NE, USA).

### 4.5. RNA Extraction and cDNA Synthesis

Frozen leaf samples (0.1 g) were crushed into a fine powder in liquid N_2_ by using a mortar and pestle. Total RNA was extracted with Trizol reagent (Tiangen Biotech Co., Ltd., Beijing, China) following the manufacturer’s protocol. RNA quality was evaluated by aspectrophotometer (NanoDrop^TM^ 1000, Thermo Fisher Scientific, Waltham, MA, USA), and genomic DNA pollution was removed by treatment with RNase-free DNaseI (Tiangen Biotech Co., Ltd., Beijing, China) at 37 °C for 30 min. Subsequently, 1 μg of purified RNA was reverse-transcribed into cDNA in 50 μL of reaction buffer with an oligo (dT) primer. The reaction buffer was incubated at 37 °C for 15 min and then terminated through heating at 95 °C for 5 min.

### 4.6. qRT–PCR

An aliquot of cDNA was used as the template for qRT–PCR analysis with the SYBR Green Real-time PCR Master Mix reagent kit (Toyobo, Osaka, Japan). qRT–PCR analysis was performed on a CFX96 system (Bio-Rad, Hercules, CA, USA) following the manufacturer’s protocol, with 20 μL of a reaction solution containing 10 μL of SYBR, 1 μL of cDNA, 1.6 μL of 10 mM primer, and 7.4 μL of H_2_O. The qRT–PCR cycling conditions were as follows: initial denaturation at 95 °C for 30 s, 40 cycles of denaturation at 95 °C for 5 s and annealing at 58 °C for 10 s, and final extension at 72 °C for 15 s. A melting curve protocol from 58 °C to 95 °C was performed to detect a single gene-specific peak for all the tested primers after the final PCR cycle reaction. Table 1 lists the specific primers of the selected genes that were designed by the online software Primer-BLAST (Available Online: http://www.ncbi.nlm.nih.gov/tools/primer-blast/). To normalize data, the *ACTIN-1* sequence was amplified as an internal reference. The relative expression levels of the tested genes were analyzed by the comparative C(T) method [40]. Average values and standard errors were calculated based on three independent biological repetitions.

### 4.7. Statistical Analysis

The data were analyzed using the *SPSS* statistical software package (SPSS Inc., Chicago, IL, USA). Statistical significances were estimated by single factor ANOVA, and all determinations were performed in three independent biological repetitions. Differences were considered significant at a probability level of *p* < 0.05. Standard deviations were marked in the figures.

## 5. Conclusions

During the grain-filling stage, Chl composition, Fm, Fv, Fv/Fm, and *Pn* in premature leaves considerably decreased, and the abilities of light energy harvesting and transferring were weakened in senescent leaves. F_0_ and 1 − Fv/Fm remarkably increased, and the expression levels of *Cab*, *PsbA*, *PsbB*, *PsbC*, and *PsbD* encoding for the reaction center of the PSII complex were significantly depressed in the mesophyll cells of senescent leaves. The damaged PSII reaction centers resulted in the weakened harvesting quantum photon and transferring light energy to PSI and PSII for carbon dioxide assimilation, leading to enhanced heat dissipation of light energy and a decrease in *Pn*, which caused a significant decrease in the seed-setting rate, 1000-grain weight, and yield per plant.

## Figures and Tables

**Table 1:** Sequence of primers for *ACTIN-1* and genes used for quantitative real-time PCR.

Gene	Primer Pairs	Products (Base Pair)
*Actin*	5′-CAGCACATTCCAGCAGATGT-3′ 5′-TAGGCCGGTTGAAAACTTTG-3′	198
*Cab*	5′-TGGCAGGACATCAAGAACCC-3′ 5′-GCTCCTTCTCCTTGGCCTC-3′	145
*PsbA*	5′-ATCTGTAGTTGATAGCCAAGGTCG-3′ 5′-TAGGTCTAGAGGGAAGTTGTGAGC-3′	118
*PsbB*	5′-ACGGTGGAGTTCTATGGTGG-3′ 5′-CCCTTGGACTGCTGCGAAA-3′	154
*PsbC*	5′-GGAGCAATGAACCTATTTGAAGTGG-3′ 5′-GCCTAAGACTGCGGAGGAAAT-3′	186
*PsbD*	5′-AACCGCAGCAGTTTCCACC-3′ 5′-CACCAACGAGTAAAATCCCCTT-3′	93

## Abbreviations

Cab	Chlorophyll a/b binding protein
Chl	Chlorophyll
*esl*	Early senescence leaf
Fm	Maximal fluorescence yield of the dark-adapted state
F_0_	Minimal fluorescence yield of the dark-adapted state
Fv	Variable fluorescence
Fv/Fm	Maximal quantum yield of PSII photochemistry
PSII	Photosystem II
*Pn*	Net photosynthetic rate
Q_A_	Primary quinone acceptor
qRT-PCR	Quantitative real-time polymerase chain reaction
